# Clinical Evaluation of Chemically Cured Conventional Glass Ionomer after Light Emitting Diode Radiant Heat Enhancement: A Randomized Controlled Clinical Trial

**DOI:** 10.4317/jced.62440

**Published:** 2025-06-01

**Authors:** Eman Awad Ebrahim, Basma Hosny Mohamed, Ola Mohamed Ibrahim Fahmy, Rehab Khalil Safy

**Affiliations:** 1 Assistant Lecturer of Conservative Dentistry, Faculty of Dentistry, Suez Canal University, Egypt; 2 Lecture of Conservative Dentistry, Faculty of Dentistry, Suez Canal University, Egypt; 3 Professor of Conservative Dentistry, Faculty of Oral and Dental Medicine, Misr International University, Egypt; 4 Associate Professor of Conservative Dentistry, Faculty of Dentistry, Suez Canal University, Egypt

## Abstract

**Background:**

Assessing the clinical efficiency of chemically cured conventional glass ionomer after light-emitting diode radiant heat enhancement using Federation Dentaire International (FDI) criteria for assessment of dental restorations, regarding both functional and biological properties immediately, after 6 months and 12 months.

**Material and Methods:**

Twenty-two healthy patients were selected where each patient had two oclusso- mesial cavities in upper or lower second permanent molar. Standardized oclusso- mesial cavities were prepared for all the selected teeth, for each patient the first tooth was restored with chemically cured conventional glass ionomer cements (GICs) without any enhancement (M1 group). Meanwhile, the second tooth was restored by the same material enhanced with radiant heat light emitting diode (LED) (M2 group). Functional and biological criteria of each restoration was clinically evaluated immediately after restoration (T0), six months later (T1), and after 12 months (T2) using Federation Dentaire International (FDI) criteria for assessment of dental restorations.

**Results:**

Chi-squared and Wilcoxon’s signed rank test revealed that there was no statistically significant difference between both groups for the tested properties at baseline and 6 months follow-up time. At 12 months of follow-up time, 55% of M1 group and 95% of M2 group were clinically successful with significant difference between them.

**Conclusions:**

Chemically cured conventional GIC enhanced with LED radiant heat exhibited better clinical performance than the same material without enhancement at 12 months of follow-up time.

** Key words:**Chemically cured glass ionomer cements (GICs), Radiant heat enhancement, Light emitting diode (LED), Randomized controlled clinical trial (RCT), Federation Dentaire International criteria for assessment of dental restorations (FDI criteria).

## Introduction

Resin composite is considered a superior material for the restoration of both anterior and posterior teeth for many decades, this could be referred to its high esthetic qualities, conservative attitude, and reinforcement of the remaining tooth structure. However multiple limitations were recorded for the resin composite material such as polymerization shrinkage with its subsequent microleakage, (1) post-operative sensitivity, marginal staining, and secondary caries are among issues that limit the use of direct restorations (2). Also resin composite is a technique-sensitive material, especially in posterior teeth that necessitates a skilled operator and a meticulous placement procedure. Its success may be jeopardized if tooth isolation or patient cooperation cannot be accomplished satisfactorily. Subsequently searching for other restorative materials that could be utilized as substitutes for resin composite materials, especially in posterior teeth, was mandatory.

One of the most used restorative materials in daily clinical practice is GICs which are characterized by several advantages such as anti-cariogenic action attributed to fluoride ion release, chemical adhesion to dental tissues, and biocompatibility (3). So GICs became the material of choice for patients with a high caries risk due to these unique characteristics (4). Unfortunately, conventional chemically cured GICs suffer from poor mechanical properties such as brittleness, surface wear, low fracture toughness, and increased sensitivity to water uptake in the early stages of setting, as a result, the use of GICs as a replacement for resin composites in posterior teeth was questionable. Therefore, multiple trials were performed to improve the mechanical properties of GICs over the years such as adding resin, reducing filler size, increasing viscosity to achieve packability, and adding reinforcement fillers (5), so they could be used in a variety of clinical applications. Although all of these modifications the ideal GICs restorative materials were not introduced. One of the suggested trials to improve the performance of GICs is the utilization of light-emitting diode radiant heat enhancement (6).

Although this novel method is involved in multiple in-vitro studies, lack of clinical studies and limited evidence-based information in the literature was found. Therefore, it was found that it will be purposive to evaluate the clinical performance of conventional GIC after light-emitting diode radiant heat enhancement using a randomized controlled clinical trial. The null hypothesis of the current study was that there is no significant effect of the LED on the clinical performance of the conventional GIC.

## Material and Methods

- Study design and sample size calculation

**Figure 1:**
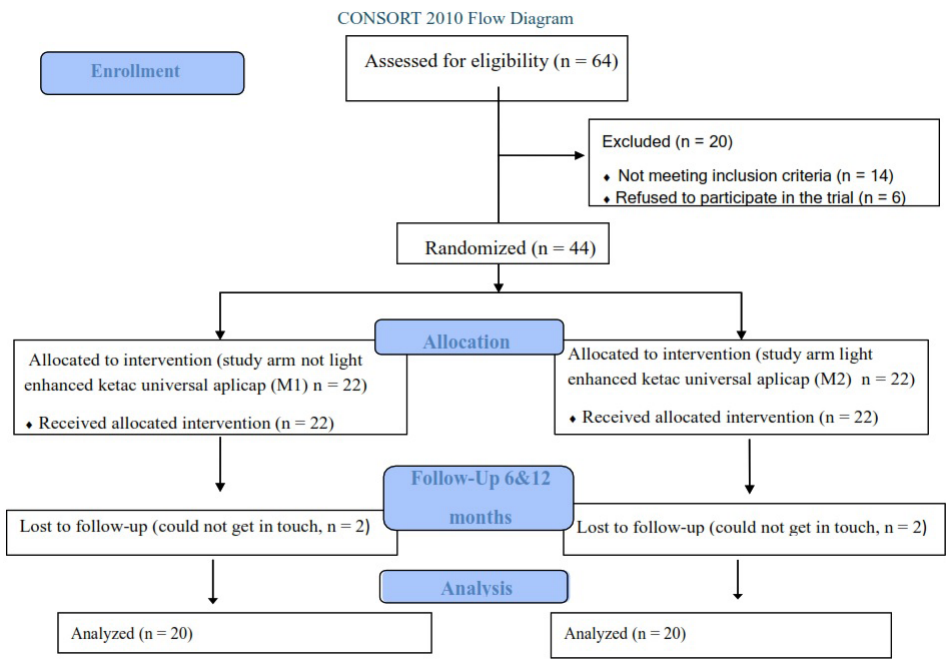
CONSORT 2010 flow diagram.

This study was performed after the approval of the Ethical Committee of Suez Canal University, Faculty of Dentistry number #431/2021, and was registered in clinical trials.gov (NCT05744622) on 27/02/2023. The present study was a double-blinded, randomized controlled clinical trial. Healthy patients with two occluso-mesial carious lesions in the upper or lower second permanent molars were selected. All patients signed an informed written consent to participate in the study after being completely aware of the benefits and potential side effects of the study. This study was reported in compliance with a protocol established by CONSORT (Consolidated Standards of Reporting Trials) guidelines (Fig. 1) (7). A sample size of 36 molars was sufficient to detect the effect size represented by 18 molars per group (8). To account for dropouts, the sample size was expanded by 20%, which produced 22 restorations for each group instead of 18 (9). The G*Power software version 3.1.9.2 was used to calculate the sample size (10).

- Inclusion and Exclusion criteria of participants and teeth:

Healthy cooperative patients were recruited from the Conservative Dentistry Department’s outpatient clinic at the Faculty of Dentistry, Suez Canal University. All selected patients were aged 25 – 40 years (11). Patients complaining from any of the following criteria were excluded from the current study, systemic diseases, clenching, bruxism, or temporomandibular joint disorders. All selected teeth were vital without any signs or symptoms of periapical pathosis or irreversible pulpitis, with healthy periodontium, in contact with neighboring teeth, and with normal occlusion. Teeth with extreme attrition or deep carious lesions (more than 2 mm depth) were all excluded from the study.

- Cavity preparation and Restorative treatment:

Restricted class II (occluso-mesial) cavity was prepared according to the manufacturer’s instructions (12), using fissure diamond bur No. #245 (Mani INC, Utsunomiya, Japan) attached to a high-speed handpiece (NSK, INC, Japan) with copious air-water coolant. A sharp excavator (N.51-52 excavator, Dentsply Maillefer international INC, UK) and large carbide round burs at low speed (ISO#500.204.001.001, Frank Dental, Gmund, Germany) were used to remove soft carious lesions. Ismuth portion for the used GIC should be less than one-half of the intercuspal distance according to the manufacturer’s instructions (12), the average bucco-lingual width of occlusal cavities was prepared approximately one-third of the inter-cuspal width, and the depth was 0.5 beyond the dentino enamel junction (13). Each case was isolated utilizing the rubber dam (GDC/ Hu Friedy, Chicago, USA). Proper proximal contour and contact with the neighboring tooth were established using a sectional matrix system (TOR VM Sectional Matrix Kit, Moscow, Russia). The type of restoration was detected through picking up of closed envelope for each tooth each time. The restorative procedure was carried out according to the manufacturer’s recommendations (12). Each cavity was carefully rinsed with copious water before being dried with air without desiccation. Cavities were classified into two groups according to the mode of enhancement of the material used (M). where M1: cavities were restored by chemically cured conventional GICs (KetacTM Universal AplicapTM, 3M ESPE, Neuss, Germany) without any mode of enhancement, meanwhile M2 cavities were restored with the same restorative material enhanced with LED radiant heat.

For restoration of the M1 group, GIC was applied according to the manufacturer’s instructions (12), then restoration was finished and polished, and any premature intervention was eliminated. Restoration of the M2 group was performed following the same steps that were carried out for the M1 group, but the achieved restoration was irradiated by LED radiant heat with a standard 1200 mW/cm² output intensity (3 M, ESPE Elipar S10) for 60 seconds, all the curing procedure was performed before any finishing and polishing step (14). Material used in the study is represented in  Table 1.

- Evaluation of clinical performance and follow-up:

Each restoration was evaluated by two qualified blind examiners. According to the FDI criteria, each restoration was assessed based on functional properties [fracture of material and retention, marginal adaptation, occlusal contour and wear, proximal contact point and food impaction, and radiographic examination] and biological criteria [postoperative sensitivity and recurrence of caries] (15). Each restoration was evaluated; Immediately after restoration placement (T0), six months later (T1), and 12 months (T2).

Evaluation of the restorations was done clinically by Bitewing radiographs and visual inspection using dental mirrors, a light source, magnification loupes (4.5x; Carl Zeiss GmbH, Jena, Germany), and FDI-recommended probes with different tip diameters of 150 and 250 μm (15) (150x and 250x, Deppeler, Switzerland). Restorations were scored using a scale ranged from 1 to 5, where score 1: clinically excellent or very good, score 2 clinically good, score 3 clinically satisfactory, score 4 clinically unsatisfactory but repairable, and score 5 clinically poor or irrepairable. Scores 1,2 and 3 were considered clinically successful while scores 4 and 5 were considered unsuccessful.

For ethical reasons, the operator took action for the cases that were scored as 4 or 5 in six months follow up (T1). For cases receiving a score of 4, restorations were repaired; for cases with a score of 5, the entire old restoration was removed and replaced with new materials. The scores of the failed restorations were maintained at levels like those obtained six months after consulting a statistician for statistical purposes.

-Statistical analysis:

Data were collected, checked, revised, and organized in Tables and Figures using Microsoft Excel 2016 and IBM-SPSS advanced statistics version 29.0 (16). Differences in evaluations between mode of restoration enhancement (M1 and M2) were carried out by Chi-squared and Wilcoxon’s signed rank test at 0.05 level. However, differences between follow-up times (T0, T1, and T2) were carried out by Friedman’s test for related samples for nonparametric data. Variations caused by both materials and follow-up times in addition to interaction between them were assessed by repeated measures ANOVA for ranked data at significance levels of 0.05.

## Results

**Figure 2:**
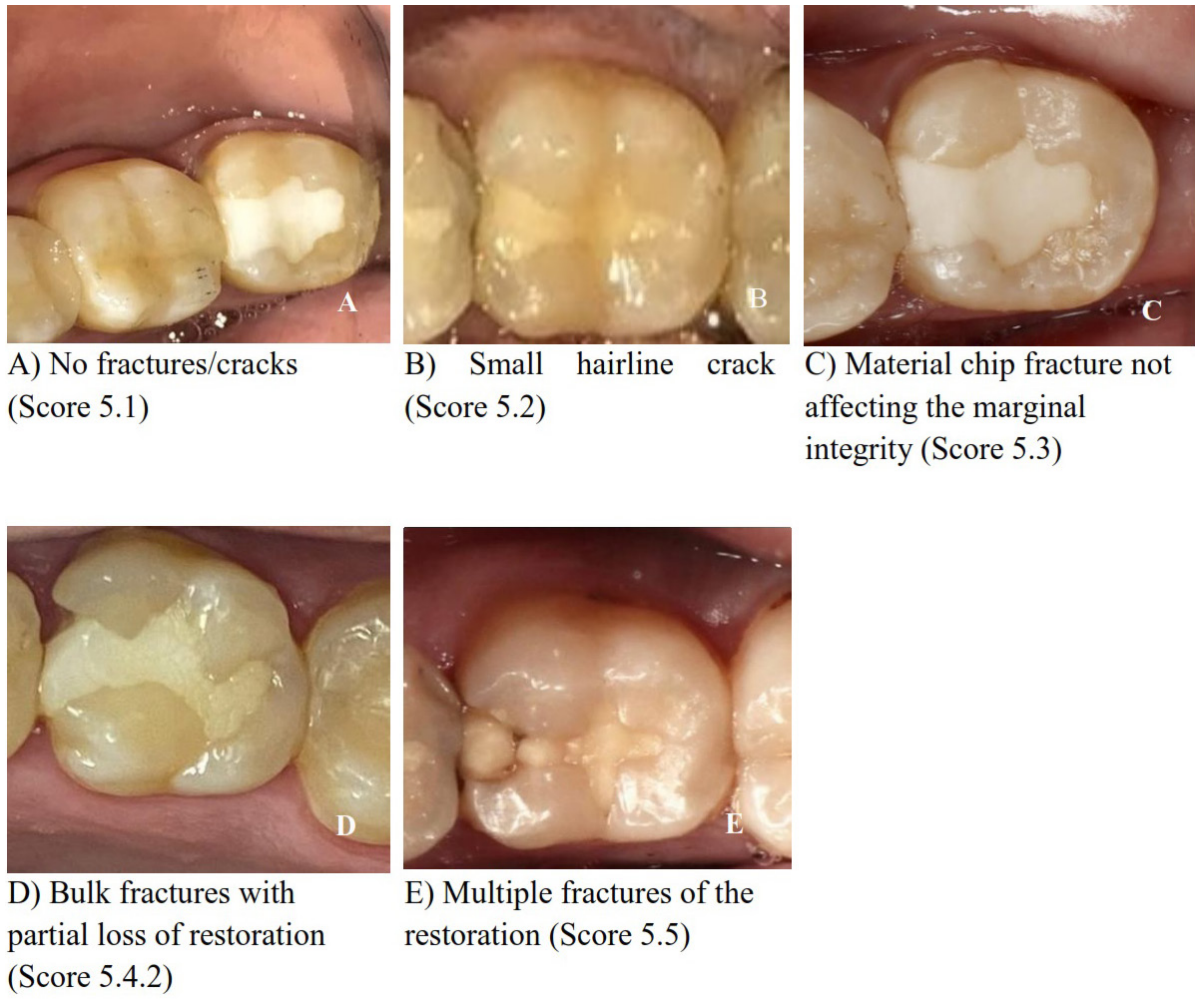
Representative photographs of the fractures and retention results. A) No fractures/cracks (Score 5.1). B) Small hairline crack (Score 5.2). C) Material chip fracture not affecting the marginal integrity (Score 5.3). D) Bulk fractures with partial loss of restoration (Score 5.4.2). E) Multiple fractures of the restoration (Score 5.5).

The overall functional and biological properties result of both tested groups (M1 & M2) at different follow-up times (T0, T1 & T2) are listed in Table 2. The results revealed that at baseline (T0), all cases (100%) of each group were clinically accepTable (score 1), with no significant difference between them. Meanwhile, at six months follow-up time (T1), 18 restorations (90%) of M1 and 20 restorations (100%) of M2 groups showed an overall collective functional and biological clinical acceptance with no significant difference between them. Meanwhile, at 12 months follow-up time (T2), 11 restorations (55%) of M1 and 19 restorations (95%) of M2 groups were clinically successful with significant differences between them. ANOVA repeated measures showed that there is a significant difference in overall functional and biological properties results induced by the two tested groups, follow-up times, and interaction between them. Representative photographs of the fractures and retention results are shown in Figure 2.

## Discussion

Thanks to their excellent aesthetic and favorable functional properties, resin composites are considered the most popular adhesive material. However, leakage with occasional postoperative sensitivity and secondary caries is often one of the main adhesive restoration failure reasons. Based on previously mentioned drawbacks, GIC has been widely researched because of its numerous benefits over other restorative materials. GIC bonds chemically to the tooth substrate, has superior anticarcinogenic efficiency and is a biocompatible material. Unfortunately, conventional chemically cured GICs suffer from poor mechanical properties as a result, GICs are still not commonly employed in restorative dentistry for permanent fillings (17). One of the suggested trials to improve the performance of GICs is the utilization of LED radiant heat enhancement.

Regarding the functional properties, fracture of material and retention as well as occlusal contour and wear results showed that at baseline all restorations of each group showed clinically excellent behavior, with no significant difference between them. However, at six months of follow-up time, 90% of the M1 group and 100% of M2 group were clinically successful with no significant differences between them. Meanwhile, at 12 months of follow-up time, the success rate of the M2 group (95%) was higher than the M1 group (55%) with significant differences between them.

This result could be attributed to the acceleration of the maturation process of GICs through shortening the vulnerable initial stage in the setting reaction by providing external energy that could be the reason for improving the physical and mechanical properties of the enhanced M2 group over the non-enhanced M2 group (18,19). Regarding the marginal adaptation results at baseline and six months, both groups were clinically successful without significant difference between them. However, the radiant heat-enhanced LED group (M2) showed significantly more successful marginal adaptation results than the non-radiant heat-enhanced LED group (M1) at 12 months. This might be attributed to the application of heat improves bond strength and decreases microleakage of GICs (20), which might be due to changes in molecular kinetic energy caused by radiant heat application that may lead to a rearrangement of the molecules in the material which increases adaptation and bond strength to enamel, providing better adhesion of the material to tooth tissues, and decrease the porosity inside the material (21).

The results of the proximal contact point and food impaction property showed that there was no significant difference between M1 and M2 at the baseline and 6 months. However, after 12 months, M2 group was clinically more successful than M1 group with significant differences between them. This could be due to the superior mechanical properties and increased marginal adaptation of the M2 group as mentioned before. Also, the applied heat transmitted through the metal matrix may have accelerated the setting time of the susceptible proximal areas (22). It is worth mentioning that the radiographic examination results were following the clinical examination findings, where at baseline and six months both groups showed acceptable results with no significant difference between them. However, after 12 months, there was a significant difference between the two groups.

Regarding the biological properties, postoperative sensitivity, and tooth vitality results showed no significant difference between both groups at baseline and after 6 months and were clinically successful without post-operative sensitivity. Meanwhile, after 12 months, the M2 group was clinically more successful than the M1 group with significant differences between them. This may be due to the lack of marginal adaptation of the M1 group as mentioned before that is associated with marginal leakage, postoperative sensitivity, and recurrence of caries, as the higher the rate of microleakage, the greater post-operative sensitivity would be. On the other hand, the good result of post-operative sensitivity of the M2 group is due to its outstanding marginal adaptation result and increased chemical bonding to the tooth structure (23).

Regarding the caries recurrence property results it revealed a statistically significant difference between the M2 and M1 after 12 months, where M2 group was clinically more successful than the M1 group. This may be due to increased fluoride release after LED radiant heat enhancement which has an anti-cariogenic effect (18). In contrast other searchers found that a reduction in fluoride release occurs after the treatments (6). This may be due to the acceleration of the initial setting of GICs by the effect of heat induced by the dental LED unit possibly reducing the burst effect of the first process of fluoride release, which takes place in the first hours after mixing. These variations may be related to the experimental variables in the in-vitro studies, such as the internal structure of the material, including the composition, geometric structure, solubility, and porosity of the material used, the powder/ liquid ratio during the preparation, fluoride content, ambient temperature, surface applications, different measurement methods (24). From all previously mentioned results, the null hypothesis of the current study was partially rejected, as there was a significant difference at 12 months follow-up between the clinical performance of conventional chemically cured GICs enhanced with LED radiant heat and the same material without any mode of enhancement in class II cavities.

## Conclusions

1- Conventional chemically cured GICs enhanced with LED radiant heat exhibited better clinical performance than the same material without any mode of enhancement when used for restoring class II cavities at 12 month.

2- Conventional chemically cured GICs without any mode of enhancement could be used as an interim restoration in stress-bearing areas in class II cavities.

3- Clinical trials with longer follow-up periods are advised to confirm the current results.

## Figures and Tables

**Table 1:** Material brand name, description, composition, batch number, and manufacturer.

Material brand name	Description	Composition	Batch number	Manufacturer
Ketac^TM^Universal Aplicap^T^^M^	Conventional chemically cured glass ionomer Shade: A2	Powder: Al-Ca-La fluorosilicate glass Liquid: Water, copolymer of acrylic acid, malic acid, tartaric acid	7726494	3M ESPE (Neuss, Germany) https://www.3MESPE.com

**Table 2:** Statistical analysis of collective functional and biological properties results.

Follow-up time (T)	Collective Functional and Biological properties				Wilcoxon's signed rank test
	M1 (Not light enhanced)		M2 (Light enhanced)		
	F	S	F	S	
Baseline (T0)	0 (0)	22 (100)	0 (0)	22 (100)	>0.05
6 months (T1)	2 (10)	18 (90)	0 (0)	20 (100)	0.602
12 months (T2)	9 (45)	11 (55)	1 (5)	19 (95)	0.030*
Friedman's sign.	0.008 **		0.317		
ANOVA -repeated measures					
Materials (M)	<0.001***				
Follow-up time	0.006**				
Materials x Time	0.038 *				

S means successful restorations; F means failed restorations

## Data Availability

The datasets used and/or analyzed during the current study are available from the corresponding author.
